# Rothe Time Propagation for Coupled Electronic and Rovibrational Quantum Dynamics

**DOI:** 10.1021/acs.jpca.5c01732

**Published:** 2025-06-09

**Authors:** Aleksander P. Woźniak, Ludwik Adamowicz, Thomas Bondo Pedersen, Simen Kvaal

**Affiliations:** † Faculty of Chemistry, 201868 University of Warsaw, Pasteura 1, 02-093 Warsaw, Poland; ‡ Department of Chemistry and Biochemistry, 8041 University of Arizona, 1306 E University Blvd, Tucson, Arizona 85721-0041, United States; § Hylleraas Centre for Quantum Molecular Sciences, Department of Chemistry, 6305 University of Oslo, P.O. Box 1033 Blindern, N-0315 Oslo, Norway

## Abstract

When time-propagating a wave packet representing a molecular system interacting with strong attosecond laser pulses, one needs to use an approach that is capable of describing intricate coupled electronic-nuclear events that require departure from the conventional adiabatic Born–Oppenheimer (BO) approximation. Hence, the propagation should be carried out simultaneously for the electrons and nuclei, treating both particle types on an equal footing *without* invoking the BO approximation. In such calculations, in order to achieve high accuracy, the wave packet needs to be expanded in basis functions that explicitly depend on interparticle distances, such as all-particle explicitly correlated Gaussians (ECGs). In our previous work, we employed basis sets consisting of ECGs with optimizable complex exponential parameters to fit time-dependent wave functions obtained from grid-based propagations of two model systems: a nucleus in a Morse potential and an electron in a central-field Coulomb-like potential, subjected to intense laser pulses. In this work, we present a proof-of-principle study of the time propagation of linear combinations of ECGs for these two models using Rothe’s method. It is shown that the approach very closely reproduces the virtually exact results of grid-based propagation for both systems. This provides further evidence that ECGs constitute a viable alternative to purely grid-based simulations of coupled nuclear-electronic dynamics driven by intense laser pulses.

## Graphical abstract

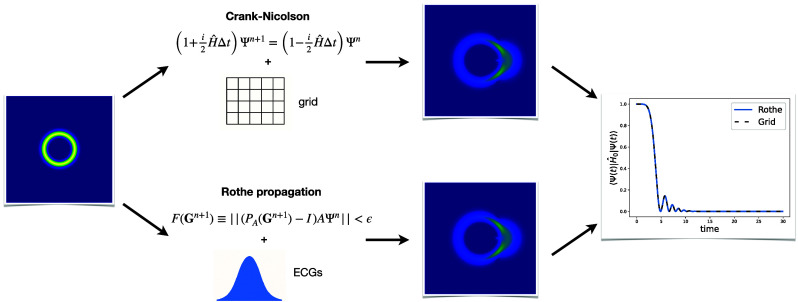

## Introduction

Experimental studies of the dynamics of chemical and physical processes at the atomistic level usually involve simulations to aid interpretation. This is particularly true for events triggered by the interaction of individual atoms, molecules, or simple clusters with high-energy photons. New experimental techniques for manipulating atoms and molecules with intense ultrashort (atto- and few-femtosecond) laser pulses has revealed the complicated nature of the dynamics of these experiments. To ensure correct interpretation of the experimental observations, reliable quantum-dynamics (QD) simulations of the experiments need to be performed. As the intense broad-band laser pulses deliver high energy to a molecule, all degrees of freedom are affected. These include motions associated with rovibrational transitions and motions associated with electronic and vibronic transitions, as well as motions resulting in ionization and/or fragmentation of the system. In all these motions the coupling of the electronic and nuclear degrees of freedom plays a significant role. Thus, QD simulations of the molecular systems interacting with ultrashort intense laser pulses carried out by integrating the time-dependent Schrödinger equation (TDSE), have to include a proper description of all these effects. In particular, the approach used has to depart from the Born–Oppenheimer (BO) approximation^1,2^ because only then can the coupling of the electronic and nuclear degrees of freedom be accurately described. This is the approach investigated in the present work.

If a finite basis set is employed in the simulation, the basis functions that are used to expand the time-evolving wave packet representing the system must be capable of describing an array of effects. These include the correlation effects associated with the Coulombic electron–electron, electron–nucleus, and nucleus–nucleus interaction of which the latter is the strongest. It happens because heavy nuclei with alike charges stay very strictly separated in their motions. This is different for the much lighter electrons. Their wave functions are wider and overlap, resulting in a noticeable probability of finding two electrons in the same point in space. The electronic-nuclear correlation is also strong, as the electron, particularly the core electrons, follow the nuclei in their motion very closely.

The other effects that the basis functions used in the QD simulation need to describe are the oscillations and axial deformation of the wave functions along the field direction, caused by rotation of the direction and oscillation of the intensity of the laser pulse. The oscillations can also be caused by the wave packet acquiring contributions from states representing a large range of the rovibrational and electronic excitations of the system.

All the above-mentioned effects necessitate the use of very flexible basis functions, whose size must be adjusted in the simulation process and whose parameters (linear and nonlinear) are thoroughly optimized at every simulation step. The basis functions used in this work and the procedure used in the laser-induced time propagation of the wave packet representing the system are described in the next section. To best describe the mentioned effects, particularly the coupling effects, using a basis-set approach, one needs to expand the wave function in terms of functions that explicitly depend on the interparticle coordinates, i.e., nucleus–nucleus, nucleus-electron, and electron–electron distances. Such functions are called explicitly correlated functions (ECFs). In the first part of this work, we review the ECFs used in the atomic and molecular calculations of stationary bound states and we discuss the features of these functions that make them particularly useful in QM calculations of atomic and molecular systems. In particular, we focus on all-particle Gaussian ECFs—explicitly correlated Gaussians (ECGs)—as these are the most popular functions used in non-BO high-accuracy atomic and molecular calculations.^3–17^ In recent years, ECGs have also been successfully used to very accurately reproduce the results of high-resolution spectroscopic experiments.^18–20^ Moreover, shifted ECGs have been shown to correctly capture impulsive laser alignment without the BO approximation.^21^

The main focus of the present work is the effectiveness of ECGs. The Gaussians used in the present work combine two features that have been proven instrumental in describing the topology of the wave function of very highly excited rovibrational states of diatomic molecules and the deformation of the highly correlated wave function of a molecule interacting with a static electric field.^6,22–24^ The mentioned features include the shifts of the centers of the ECGs away from the origin of the internal coordinate system and making both the elements of the matrix of the exponential parameters of the Gaussians and the elements of the vector of the shifts to be complex numbers. These extensions allow us to describe the radial and angular oscillations of the wave function. It also allows us to describe the interparticle correlation, in particular the strong internuclear correlation, as it was shown previously for a diatomic system.^25–28^ The complex shift coordinates together with the complex exponential parameters enable one to describe dissociation and fragmentation processes. In the present work, we do not use complex shifts, but instead an equivalent parametrization in terms of real shifts and plane-wave factors (see the next section).

Single-particle Gaussians have been extensively used as basis functions in both electronic-structure theory^29^ and vibrational dynamics.^30–32^ In electronic-structure theory the Gaussians are real-valued functions centered at the atomic nuclei and contracted to form atomic orbitals which, in turn, form a nonorthogonal basis for the expansion of molecular orbitals—see, e.g., ref 29. for a detailed account.

Single-particle Gaussians have been used before to study molecular vibrational dynamics^30–32^ multielectron dynamics.^33–35^ In the vibrational-dynamics approach proposed by Heller, the use of complex-valued Gaussians, was motivated by these functions being exact solutions for harmonic potentials. In this approach, the Gaussian parameters are time-dependent variational parameters. However, when the potential becomes anharmonic, the time-dependent equations of motion quickly become ill-behaved and one needs to resort to locally harmonic approximations.^36^ One should also mention the use of single-particle complex-valued Gaussians in the multiconfigurational time-dependent Hartree (MCTDH) method.^37–40^ However, also in this case, ill-conditioned equations arise that hamper the use of the method.

Restricting the Gaussians to real-valued functions does not allow to describe such important highly nonlinear phenomena as ionization and high harmonic generation processes. Such processes are crucial in the dynamics involving short and extreme laser pulses. Some attempts have been made to alleviate this problem by augmenting regular, non-correlated Gaussians with functions capable of representing the continuum have been made by Coccia and Luppi,^41^ but this approach has found limited application so far.

To test the ability of ECGs to describe molecular QD, in our previous work,^43^ we used the virtually exact numerical wave packets determined on a grid to fit linear combinations of ECGs. The fitting was done for two two-dimensional (2D) models representing laser-induced single-particle dynamics occurring in a Coulomb potential and in a Morse potential. These two dynamics are essential elements of the dynamics that occur when a diatomic molecule is interacting with laser pulses, in particular, with large-frequency and high-intensity ultrashort laser pulses. The tests were relevant to laser-induced dynamics of attosecond atomic and molecular events involved in attosecond experiments.^44–46^

In the present work, the testing of the ability of the ECGs to represent time-evolving wave packets in the two previously considered 2D models is extended to generating these packets by solving the time-dependent Schrödinger equation using a novel approach called the Rothe time-propagation method,^47–51^ in which discretization is done *first in time*, leading to each time step being an optimization problem for finding the correct wave function in terms of the nonlinear parameters of the ECGs, as well as the linear expansion coefficients. The Rothe time-propagation method has been studied previously on the high-harmonic generation from a hydrogen atom and on dynamics in the Henon–Heiles potential,^50,51^ showing encouraging results. The present study is complementary, focusing on 2D model systems in preparation for future studies of multiparticle systems without the Born–Oppenheimer approximation.

Unlike standard post-BO approaches in molecular QD, which commonly employ the Born-Huang ansatz and rely on potential energy surfaces (PESs) derived from electronic structure calculations, our approach does not assume the BO approximation from the outset. In traditional methods, the wave packet is propagated on a small number of PESs, either using an average force field or some model for interstate hopping. This is often reasonable when the system remains bound and does not undergo ionization or dissociation. However, attosecond laser pulses are inherently broadband, making ionization processes virtually unavoidable and preselection of a limited number of excited-state (neutral and ionized) PESs practically impossible. Near dissociation and ionization thresholds, many excited electronic states may contribute, each associated with a distinct PES. Furthermore, as the energy gaps between electronic and nuclear states narrow near these limits, strong nonadiabatic couplings emerge, often invalidating the BO approximation altogether. In such scenarios, a direct non-BO method like the one used here becomes essential.

A natural question arises about the choice of propagation technique. The most widely used propagation methods are based on the Dirac-Frenkel variational principle which projects the Schrödinger equation onto the tangent manifold defined by the wave packet ansatz. It is well-known, however, that this approach suffers from severe numerical instabilities when applied to linear combinations of Gaussians with adjustable parameters. In this work, we avoid these instabilities by using Rothe’s method where a reversal of temporal and spatial discretizations converts the propagation into an optimization problem in each time step. In addition to improved numerical stability, Rothe’s method allows us to reparameterize the wave packet ansatz, including changing the number of basis functions, in each time step and thus provides extreme adaptivity. This is a crucial feature of Rothe’s method in the context of complicated nonadiabatic quantum dynamics.

The presentation of this work starts with a description of the Hamiltonians used in the calculations. Next, the Rothe time-propagation method is briefly outlined. This is followed by a section on the ECGs used in the calculations and a section describing the results of the test calculations. Lastly, the main results of this work are summarized.

## Theory and Methods

### Non-BO Hamiltonian of the Molecular System

The aim of this work is to develop a procedure for solving the time-dependent Schrödinger equation (TDSE), iψ̇(*t*) – *Ĥ*(*t*)­ψ­(*t*) = 0, for a molecular system interacting with a short but intense laser pulse. The external laser field is treated within the electric-dipole approximation, i.e., we neglect the spatial variation of the electric field and omit the magnetic component. The electric-dipole approximation captures the leading effects also in strong laser fields provided that the wavelength is much greater than the spatial extent of the electronic wave function, which holds even at high intensities for typical optical frequencies used in attosecond experiments.^52,53^ Moreover, going beyond the electric-dipole approximation would require us to take into account the coupling of the electromagnetic field to the center-of-mass motion, which is beyond the scope of the present work. The total nonrelativistic all-particle non-BO molecular Hamiltonian describing the interaction of a neutral molecule with an electric field with time dependent strength oriented along a chosen direction (we choose the direction along the *x*-axis of a laboratory-fixed coordinate frame) can, in the dipole approximation, be rigorously separated into a center-of-mass (COM) kinetic-energy operator and the internal Hamiltonian,^3,5,54^ *Ĥ*(*t*). The separation is performed by transforming the total lab-frame Hamiltonian from the Cartesian laboratory coordinates, **R**_*i*_, *i* = 1, ..., *N*, where *N* is the total number of particles in the molecule, to a new Cartesian coordinate system, whose first three coordinates are the lab-frame coordinates of the COM and the remaining coordinates are internal coordinates. The internal coordinate axes are parallel to the lab-frame axes and have their origins located at a selected particle called the reference particle (particle no. 1; it is usually the heaviest nucleus in the molecule). The internal coordinates, {***r***_*i*_}, are ***r***_*i*_ = ***R***_*i*+1_ – ***R***_1_ where *i* = 1, ..., *n* and *n* = *N* – 1. The internal Hamiltonian only depends on the internal coordinates and have the following form^3^ (we use atomic units throughout):

1 $$Ĥ\left(t\right)=\sum_{i=1}^{n}\left(-\frac{1}{2\mu_{i}}\nabla_{r_{i}}^{2}+\frac{q_{0}q_{i}}{r_{i}}\right)+\sum_{i<j}^{n}\left(\frac{q_{i}q_{j}}{r_{\mathrm{ij}}}+\frac{1}{M_{1}}\nabla_{r_{i}}^{\prime }\nabla_{r_{j}}\right)-F\left(t\right)\sum_{i=1}^{n}q_{i}x_{i}$$

where F(t) is the time-dependent electric-field strength, *M*_1_ is the mass of the reference particle (particle 1), *q*_*i*_ = *Q*_*i*+1_ (*i* = 0, ..., *n*), μ_*i*_ = *M*_1_ *M*_*i*+1_/(*M*_1_ + *M*_*i*+1_) is the reduced mass of particle *i* (*i* = 1, ..., *n*), *Q*_*i*_ and *M*_*i*_ are the charge and mass of particle *i* (*i* = 1, ..., *N*), respectively, *r*_*ij*_ = |**r**_*i*_ – **r**_*j*_| = |**R**_*i*+1_ – **R**_*j*+1_|, *r*_*i*_ = |**r**_*i*_ |, and the prime denotes vector/matrix transposition. As one notices, internal Hamiltonian ([Disp-formula eq1]) represents *n* interacting particles with masses equal to the reduced masses moving in the central Coulomb potential of the reference particle. We refer to the particles, whose total internal energy is described by the internal Hamiltonian, ([Disp-formula eq1]), as *pseudoparticles* because, while they have the same charges as the original particles, their masses are changed to the reduced masses. For F(t)=0 , the Hamiltonian ([Disp-formula eq1]) is fully symmetric (isotropic or atom-like) with respect to all rotations around the center of the internal coordinate system. Thus, the eigenfunctions of ([Disp-formula eq1]) transform as irreducible representations of the fully symmetric group of rotations. However, when F(t)≠0 , the symmetry of ([Disp-formula eq1]) is reduced to cylindrical with the symmetry axis being the field direction (here the *x*-axis). Also, the appropriate permutational symmetry for each group of identical particles in the system needs to be implemented in the eigenfunction. It may involve (for example, as it does in the case of the H_2_ molecule) the reference particle.

### Grid-Based Calculations of 2D Model Systems

In the internal Hamiltonian ([Disp-formula eq1]) for a diatomic system, the potential acting on the second nucleus that results from the interaction of this nucleus with the charge of the reference nucleus and the electrons can effectively be represented by a Morse-like potential. An important effect that also determines the electronic-nuclear dynamics of the system is the electrostatic attractive interaction of each of the electrons with the reference nucleus located at the center of the internal coordinate system. Thus, the dynamics of an electron interacting with a central potential created by the charge of the reference nucleus and the dynamics of a particle interacting with a Morse potential are central to understanding the laser-induced dynamics of a diatomic molecule. The dynamics of these two models is investigated in 2D in the present work.

As a near-exact numerical reference for the Rothe propagations performed in this work, we utilize the results of grid-based simulations of the Morse model and of the Coulomb model presented in our previous paper, ref 43. The detailed methodology and the justification for the choice of the simulation parameters are provided there, while here we only summarize the key assumptions of the calculations for the sake of completeness. For both 2D models we use a Hamiltonian of the form:

2 $$Ĥ\left(t\right)=-\frac{1}{2\mu }\left(\frac{\partial^{2}}{\partial x^{2}}+\frac{\partial^{2}}{\partial y^{2}}\right)+V\left(x,y\right)-\mathrm{qx}F\left(t\right)$$

where for the electron we use the soft Coulomb potential, *V*(*x*, *y*) = – (*x* ^2^ + *y* ^2^ + 1/2)^−1/2^, which mimics the nuclear potential of a hydrogen-like atom, and for the Morse potential we use *V*(*x*, *y*) = *D*_*e*_ [1 – exp (−α ((*x* ^2^ + *y* ^2^)^1/2^ – *r*_*e*_))]^2^, with *D*_*e*_ = 0.17449, *r*_*e*_ = 1.4011, and α = 1.4556. The charge and (reduced) mass are set to *q* = – 1, μ = 1 for the Coulomb model, and *q* = 1, μ = 1605.587 for the Morse model. The laser pulse is represented by a cosine wave with a trigonometric envelope.^55^ The electric field is nonzero only in the time interval *t*_0_ < *t* < *t*_1_, where it is equal to

3 $$F\left(t\right)=E_{0}{\;\sin}^{2}\left(\pi \frac{t-t_{0}}{t_{1}-t_{0}}\right)\;\cos\left(\omega \left(t-\mathrm{t̅}\right)\right),\mathrm{t̅}=\frac{t_{0}+t_{1}}{2}$$

where $E_{0}$ denotes the maximum electric field amplitude. In our calculations for both models we set *t*_0_ = 0. For the Coulomb model we set ω = 0.25 au, *t*_1_ = 60 au and $E_{0}=0.4$ a.u., which corresponds to a laser pulse of wavelength ≈ 182 nm, with a duration of 2.5 optical cycles (foot-to-foot duration 1.45 fs) and a peak intensity of 5.6 × 10^15^ W/cm^2^. For the Morse model we set ω = 0.0 au, *t*_1_ = 20 au (0.48 fs) and $E_{0}=2.0$ a.u., corresponding to a short, delta-like pulse which violently but very briefly pushes the particle in the positive *x*-direction. We consider the dynamics for times 0 ≤ *t* ≤ 100 au for the Coulomb model and 0 ≤ *t* ≤ 300 au for the Morse model, including periods of free evolution after the laser pulse. The time-dependent wave functions are discretized on a spatial grid consisting of *n*_grid_ = 1024 equidistant points in the interval [−*L*, *L*] = [−150, 150] for the Coulomb model and [−20, 20] for the Morse model, in both spatial directions. These intervals are found to be sufficient to ensure that the wave function does not reach the grid boundaries before the end of the simulation. We employ the Fourier pseudospectral method for discretization of the Hamiltonian.^56^ The ground state wave functions are determined by minimizing the expectation values of the respective field-free Hamiltonians using the conjugate gradient method with inverse iterations. In case of the Coulomb system this results in a 2D 1*s* orbital, while for the Morse potential it yields an annular-shaped wave function with a peak at *r*_*e*_, i.e., at the ground-state equilibrium internuclear distance. The further time-propagation is performed using the second-order split-operator approach. The adopted time step is *h* = 0.01 au for the Coulomb model and *h* = 0.05 au for the Morse model. Some snapshots of the time evolution of the wave functions for the Coulomb and Morse simulations featured in this work can be found in ref 43. In both cases the respective wave functions become increasingly more complicated with many features, more deformed and oscillatory, and more diffused as the time-simulation progresses.

### Fully Flexible ECG Basis Set

As elaborated in our previous work,^43^ complex ECGs with optimizable nonlinear parameters are the building blocks of the time-dependent wave function in our approach. The Adamowicz group has used various types of ECGs to perform very accurate calculations of stationary atomic and molecular bound states for over two decades.^3–6^ A brief review of these different kinds of basis sets can be found in our previous work.^5^ Here, we employ the same form of the 2D fully flexible ECGs (FFECGs) as in our previous paper,^43^ depending on six real nonlinear parameters **G** = (*q*_*x*_, *q*_*y*_, *p*_*x*_, *p*_*y*_, *a*, *b*), i.e.,

4 $$\phi \left(r;G\right)=\exp\left[-\left(a+\mathrm{ib}\right)∥r-q{∥}^{2}+\mathrm{ip}\cdot \left(r-q\right)\right]$$

The set of FFECGs span the Hilbert space of square-integrable functions on $R^{3}$ provided that *a* > 0, which we ensure throughout the simulations. When *b* = 0 and **p** = **0**, the FFECGs reduce to ordinary Gaussian basis functions centered at **q** which are suitable for describing bound states. For small *a* and nonzero *b* and/or **p**, the FFECGs become delocalized and highly oscillatory, allowing them to effectively capture unbound states while remaining in the space of square-integrable functions.

As we demonstrated by fitting the time-dependent wave functions of the model systems, such FFECGs are very efficient in reproducing various wave function shapes, including highly complicated, oscillatory ones. On the other hand, they can also easily approximate the purely real-valued ground state wave functions, which makes them a good starting point for the time-propagation. In particular, the ground state of the Coulomb system centered at *x* = *y* = 0 consists solely of simple spherical Gaussians with *a* ≠ 0 and *b* = *p*_*x*_ = *p*_*y*_ = *q*_*x*_ = *q*_*y*_ = 0. For the Morse-model ground state, the FFECGs with *a* ≠ 0, *b* ≠ 0, and *p*_*x*_ = *p*_*y*_ = *q*_*x*_ = *q*_*y*_ = 0 need to be employed to reproduce the annular shape of its wave function. However, for the function to remain real, for each value of *a* two FFECGs with *b* and – *b* are used, so that their linear combination results in a Gaussian function multiplied by a sin (or cos) function:

5 $$\frac{\phi \left(-b\right)-\phi \left(+b\right)}{2i}=\exp\left\{-a\left(x^{2}+y^{2}\right)\;\sin\left[b\left(x^{2}+y^{2}\right)\right]\right\}=\exp\left(-ar^{2}\right)\;\sin\left(br^{2}\right)$$

As function ([Disp-formula eq5]) is zero at the center of the coordinate system, it helps to properly represent the behavior of the Morse ground-state wave function at this point. Thus, in the expanding the ground-state wave function for the Morse model, which a torus shape, the FFECGs with *a* ≠ 0, *b* ≠ 0, and *p*_*x*_ = *p*_*y*_ = *q*_*x*_ = *q*_*y*_ = 0 are used.

### Rothe Variational Method for Solving the TDSE

The spatial part of the wave function of the system, Ψ, is expanded in terms of the symmetry-adapted FFECG basis functions, ϕ_*i*_(**G**_*i*_). Each function ([Disp-formula eq4]) depends on a set **G**_*i*_ of six nonlinear parameters *a_i_*, *b_i_*, *q*_*xi*_, *q*_*yi*_, *p*_*xi*_, and *p*_*yi*_. These parameters are the matrix elements of **A** and **B** and the elements of the **p** and **q** vectors. Let us write the spatial part of the wave function as a linear combination of the FFECGs, suppressing the spatial dependence for simplicity:

6 $$\Psi \left(C,G\right)=\sum_{i}\phi_{i}\left(G_{i}\right)c_{i}\equiv \phi \left(G\right)C$$

where **C** = {*c*_*i*_} is the vector of linear expansion coefficients, and where **G** = {**G**_*i*_} is the collection of all nonlinear parameters, **G**_*i*_ being the parameters for the function ϕ_*i*_. We arrange the ϕ_*i*_ in a row “matrix” ϕ­(**G**). Using the implicit midpoint method with time step Δ*t* to discretize the TDSE *in time only* gives a space-continuous Crank–Nicolson scheme,

7 $$i\Psi^{n+1}-i\Psi^{n}-\frac{1}{2}\left(Ĥ\left(t+\frac{1}{2}\Delta t\right)\Psi^{n+1}+Ĥ\left(t+\frac{1}{2}\Delta t\right)\Psi^{n}\right)\Delta t=0$$

where Ψ^*n*^ is the time-discrete wave function at time *t* = *n*Δ*t*. Given any square-integrable Ψ^*n*^, the next time-step Ψ^*n*+1^ exists, since the solution operator is a unitary operator defined everywhere in Hilbert space.

The Rothe method for propagating the TDSE now posits an ansatz Ψ^*n*^ = Ψ­(**C** ^*n*^, **G** ^*n*^) for all time steps *n*, and variationally optimizes the residual in the semidiscrete TDSE to within a selected error threshold ϵ. We obtain the following optimization problem: Find (**G** ^*n*+1^, **C** ^*n*+1^) such that

8 $$∥A\Psi \left(G^{n+1},C^{n+1}\right)-A^{†}\Psi \left(G^{n},C^{n}\right)∥<\epsilon$$

Here, $A=I+\frac{i\Delta t}{2}Ĥ\left(t+\Delta t/2\right)$ . If the numerical optimization is not able to meet the desired tolerance ϵ, the length of the FFECG expansion is increased, allowing more details in the wave function to be resolved and thus lowering the residual. We return to the precise specification of this procedure below.

Since **C** ^*n*+1^ appears in a linear manner, we can use the standard normal equation approach (the term “standard normal equation approach” refers to an analytical method to find the linear coefficients that minimize the cost function in the ordinary least-squares minimization) to solve for these, given a current guess for **G** ^*n*+1^. This is referred to as the *variable projection method*.^57^ The solution reads

9 $$C^{n+1}=S_{A}^{-1}\phi^{†}A^{†}\Psi^{n}$$

where a weighted overlap matrix has been introduced, with elements

10 $${\left(S_{A}\right)}_{\mathrm{ij}}=\left\langle \phi_{i}\right|A^{†}A\left|\phi_{j}\right\rangle$$

Furthermore, for any wave function Φ, we have ϕ^†^Φ = **b**, with *b*_*i*_ = ⟨ϕ_*i*_|Φ⟩, a vector of length equal to the number of FFECG basis functions. We introduce an orthogonal projection operator given as

11 $$P_{A}=\left(A\phi \right)S_{A}^{-1}{\left(A\phi \right)}^{†}=\sum_{\mathrm{ij}}A\left|\phi_{i}\right\rangle {\left(S_{A}^{-1}\right)}_{\mathrm{ij}}\left\langle \phi_{j}\right|A^{†}$$

The optimization problem now reduces to: Find **G** ^n+1^ such that

12 $$F\left(G^{n+1}\right)\equiv ∥\left(P_{A}\left(G^{n+1}\right)-I\right)A\Psi^{n}∥<\epsilon$$

We now use the Gauss–Newton method to minimize the Rothe cost function *F*. The minimization is carried out as long as it takes to lower the value of *F*(**x** ^*n*+1^) below the threshold, increasing the expansion length as needed. In the result section, different values of ϵ are used in the time-propagation to test how the accuracy of the results (as compared with the results of the grid calculation) depends on the value of the threshold.

## Computational Details

The calculations involving wave packet time propagation is performed for two 2D models mentioned before. In 2D, the FFECGs are given by eq 4. Each 2D FFECG depends on six real nonlinear parameters, *a*, *b*, *p*_*x*_, *p*_*y*_, *q*_*x*_, and *q*_*y*_. As the time-propagation calculation for both models are initiated with the corresponding ground-state wave functions, which are real and spherically symmetric, appropriate FFECGs need to be used in the wave function expansion. For the Coulomb model, the FFECGs are simple spherical Gaussians centered at *x* = *y* = 0 with *a* ≠ 0 and *b* = *p*_*x*_ = *p*_*y*_ = *q*_*x*_ = *q*_*y*_ = 0. A linear combination of FFECGs of this type should provide a very accurate representation of the ground-state wave function. The linear and nonlinear parameters of FFECGs for such a representation are obtained in the present work with the use of the variational method. The minimization of the total energy of the system with respect to the linear and nonlinear FFECGs parameters results is a compact wave function that is used as a starting point for the time-propagation.

The minimization of the Rothe functional that involves optimization of all linear and nonlinear parameters of the enlarged FFECG basis set continues for some number of time steps until it is determined that the optimization process is unsuccessful because the accuracy threshold cannot be met. At that point new FFECGs are added to the basis set and the optimization continues.

The accuracy of the results obtained from the Rothe-propagation calculations for the Coulomb and Morse models can also be verified by calculating some other properties than the energy using the wave packets obtained for some selected time steps and compare of their time-dependent dynamics with the dynamics obtained using the grid method. The properties calculated in this work include the following: the expectation value of the field-free Hamiltonian, the ground-state survival probability (i.e., the square of the autocorrelation function), the dipole moment expectation value, and the expectation value of the squared *z*-component of the angular momentum. Thus, we evaluate ⟨Ψ­(*t*)|*Ĥ*_0_|Ψ­(*t*)⟩, ⟨Ψ(0)|Ψ­(*t*)⟩, ⟨Ψ­(*t*)|*x̂*|Ψ­(*t*)⟩, and ⟨Ψ­(*t*)|*L̂*_*z*_^2^|Ψ­(*t*)⟩ during time evolution.

The first steps in the FFECG calculations involves determining the nonlinear parameters that define the ground state wave functions of the two model systems, as described in the earlier subsection. In our previous work, this was done by fitting a predefined number of Gaussians to the grid-based ground states. However, here we follow a different approach, namely for both Morse and Coulomb system we explicitly optimize the ground-state Hamiltonian eigenvalue:

13 $$∥{Ĥ}_{0}\Psi \left(G^{0},C^{0}\right)-E_{0}\Psi \left(G^{0},C^{0}\right)∥=\min!$$

The minimization is carried out with respect to both linear and nonlinear parameters (*a* in case of the Coulomb model and |*b*|in case of the Morse model). There are two reasons behind this. First, by doing so, we decouple the Rothe simulations from the grid-based simulations, allowing the grid and Gaussian wave functions to evolve completely independently of each other. Second, due to numerical differences between the grid and Gaussian representation of the wave function, the ECG ground state obtained by fitting may differ slightly from the state with optimal *E*_0_. Since the Rothe method relies heavily on the minimization of the Hamiltonian value, any deviation from the actual ground state energy value may then be propagated during the time evolution. For the Coulomb model we use six FFECGs for the ground state and arrive at *E*_0_ = −0.6554858 Ha vs the numerical value of −0.6554864 Ha obtained from the grid reference calculation. For the Morse model we use eight FFECGs, obtaining *E*_0_ = −0.1639631 Ha vs the numerical value of −0.1639638 Ha.

In principle, constructing the Rothe functional requires the calculations of the matrix element of the squared Hamiltonian. This presents certain challenges, particularly in evaluating matrix elements involving products of the kinetic and potential energy operators. In this work, however, we employ an auxiliary grid-based method to discretize the product *A* ^†^Ψ­(**G** ^*n*^, **C** ^*n*^) on a two-dimensional grid. This discretized function is then used as the target in the optimization of the nonlinear parameters in *AΨ*(**G** ^*n*+1^, **C** ^*n*+1^). This approach allows us to directly minimize the cost function ([Disp-formula eq8]), thereby avoiding the need to compute squared Hamiltonian matrix elements required in solving eq 12. The minimization is carried out using the standard least-squares method as implemented in our in-house code, which utilizes the SciPy Python library.^58^ The necessary Hamiltonian and overlap matrix elements are also evaluated by first transforming the Gaussians to their grid representations, followed by numerical integration. This is a similar approach as the one used before in a development involving Slater orbitals and grid-based molecular orbitals for diatomic systems.^59^ To guarantee square-integrability of the basis functions, the square roots of the parameters *a* in eq 4 are optimized rather than *a* directly. Finally, the linear parameters **C** ^*n*+1^ are obtained by projecting the newly optimized basis set onto *A* ^†^Ψ­(**G** ^*n*^, **C** ^*n*^).

It should nevertheless be noted that although eq 12 is not explicitly used in this study, it will need to be solved in future calculations performed for a real atomic or molecular system with Gaussian functions–particularly in scenarios where the grid-based approach becomes impractical, such as systems with a larger number of particles or in higher dimensions. In previous works, we have addressed similar difficulties in the context of operators representing leading relativistic corrections, such as the mass-velocity operator, which bears resemblance to terms appearing in the square of the Hamiltonian. That experience provides a useful foundation for developing algorithms to compute the squared Hamiltonian matrix. An alternative approach we are currently exploring involves approximating the Coulomb one- and two-particle operators using linear combinations of Gaussian orbitals and Gaussian geminals (i.e., two-electron explicitly correlated Gaussians) fitted to the respective Coulomb potentials. As the coupled nuclear-electronic dynamics is likely rather insignificantly affected by particles’ near coalescent behavior, inaccuracies of the Gaussian fits of the Coulomb potentials may quite adequately describe laser-induced dynamics of small atomic and molecular systems.

At the starting point, the FFECGs are the same as used to expand the ground-state wave function but the parameters that are set to zero for the ground-state wave function are now unfrozen and become optimized. At each time step the current value of the cost function is monitored. If at some point it exceeds a predefined threshold value, new Gaussian functions are added to the basis set and the total wave function is reoptimized. This procedure is repeated until the cost function decreases below the threshold value. The choice of the initial parameters of the added basis functions is subjective, but we applied a following scheme, which we found to be quite effective. For the Coulomb model simulations we add a single FFECG at a time, with the nonlinear parameters (denoted with ′) being the averages of the respective nonlinear parameter values already present in the basis set:

14 $$\phi \left(a^{\prime }=\frac{\sum_{i}c_{i}a_{i}}{\sum_{i}c_{i}},b^{\prime }=\frac{\sum_{i}c_{i}b_{i}}{\sum_{i}c_{i}},p_{x}=\frac{\sum_{i}c_{i}p_{x,i}}{\sum_{i}c_{i}},p_{y}=\frac{\sum_{i}c_{i}p_{y,i}}{\sum_{i}c_{i}},q_{x}=\frac{\sum_{i}c_{i}q_{x,i}}{\sum_{i}c_{i}},q_{y}=\frac{\sum_{i}c_{i}q_{y,i}}{\sum_{i}c_{i}}\right)$$

For the Morse model we use a similar approach, except that we add two FFECGs at a time, with *b* ^′^s being the weighted averages of either positive or negative values of *b* in the basis set:

15 $$\begin{array}{l} \phi \left(a^{\prime }=\frac{\sum_{i}c_{i}a_{i}}{\sum_{i}c_{i}},b^{\prime }=\frac{\sum_{i,b_{i}>0}c_{i}b_{i}}{\sum_{i,b_{i}>0}c_{i}},p_{x}=\frac{\sum_{i}c_{i}p_{x,i}}{\sum_{i}c_{i}},p_{y}=\frac{\sum_{i}c_{i}p_{y,i}}{\sum_{i}c_{i}},q_{x}=\frac{\sum_{i}c_{i}q_{x,i}}{\sum_{i}c_{i}},q_{y}=\frac{\sum_{i}c_{i}q_{y,i}}{\sum_{i}c_{i}}\right)\\ \phi \left(a^{\prime }=\frac{\sum_{i}c_{i}a_{i}}{\sum_{i}c_{i}},b^{\prime }=\frac{\sum_{i,b_{i}<0}c_{i}b_{i}}{\sum_{i,b_{i}<0}c_{i}},p_{x}=\frac{\sum_{i}c_{i}p_{x,i}}{\sum_{i}c_{i}},p_{y}=\frac{\sum_{i}c_{i}p_{y,i}}{\sum_{i}c_{i}},q_{x}=\frac{\sum_{i}c_{i}q_{x,i}}{\sum_{i}c_{i}},q_{y}=\frac{\sum_{i}c_{i}q_{y,i}}{\sum_{i}c_{i}}\right) \end{array}$$

This is to ensure that the added functions reflect the (possibly distorted) annular shape of the rovibrational wave function. For both models we test three cost-function threshold values: 10^–3^, 10^–4^, and 10^–5^, for each value we monitor the value of the cost function, the error value (measured as the integral of the difference between the reference grid wave function and the Gaussian wave function), and the number of FFECGs in the basis set.

Initially, we attempted to conduct the Rothe propagations using the same time steps as in the grid-based propagations. However, we found the simulations to be numerically unstable, with the minimization procedure failing when the external field reached larger values. Consequently, the time steps are reduced to 0.002 au for the Coulomb model and to 0.01 au for the Morse model.

## Results

The results of the simulations performed in this work are presented in Figures 1–6. In Figures 1 and 2, the trajectories of four time-resolved observables are shown. These are the expectation value of the ground-state Hamiltonian, the projection of the time-dependent wave function on the respective ground state, the dipole-moment expectation value, and expectation value of the squared angular-momentum projection onto the axis perpendicular to the simulation plane. Figure 3 and 4 display arbitrarily selected snapshots from the wave function trajectories of the Coulomb and Morse models obtained from grid-based propagation and the Rothe propagation with three different cost-function thresholds. Finally, in Figures 5 and 6, the time-resolved cost function, the wave function error, and basis set size are shown.

**Figure 1:**
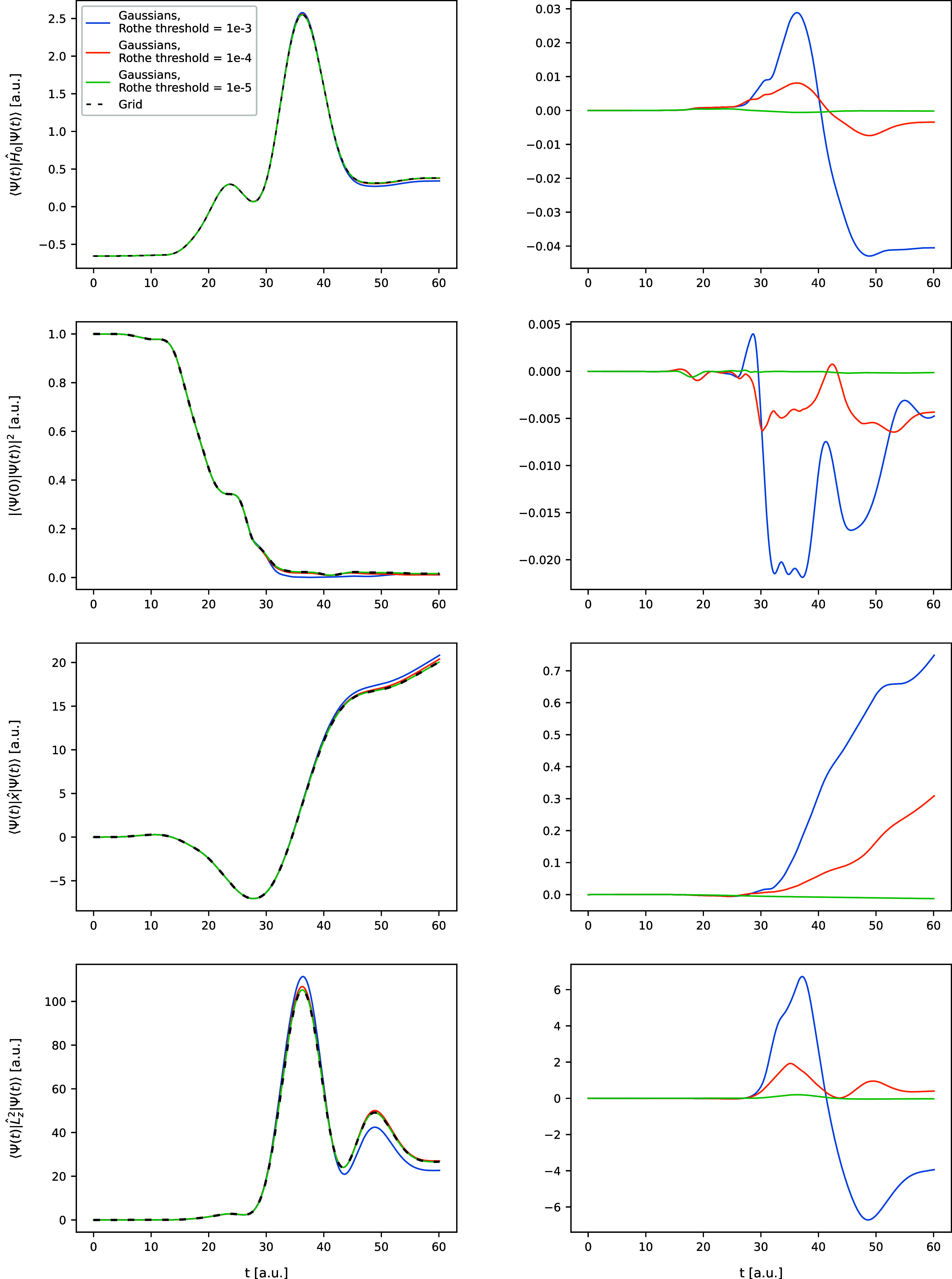
Results of the Rothe time propagation that employs FFECG basis for the Coulomb model. The plotted results concern the following expectation values: ⟨Ψ­(*t*)|*Ĥ*_0_|Ψ­(*t*)⟩, ⟨Ψ(0)|Ψ­(*t*)⟩, ⟨Ψ­(*t*)|*x̂*|Ψ­(*t*)⟩, and ⟨Ψ­(*t*)|*L̂*_*z*_^2^|Ψ­(*t*)⟩. The plots on the left for each model show the expectation values as functions of time for three values of ϵ. The plots on the right show the error with respect to the exact expectation values obtain using the grid method.

**Figure 2:**
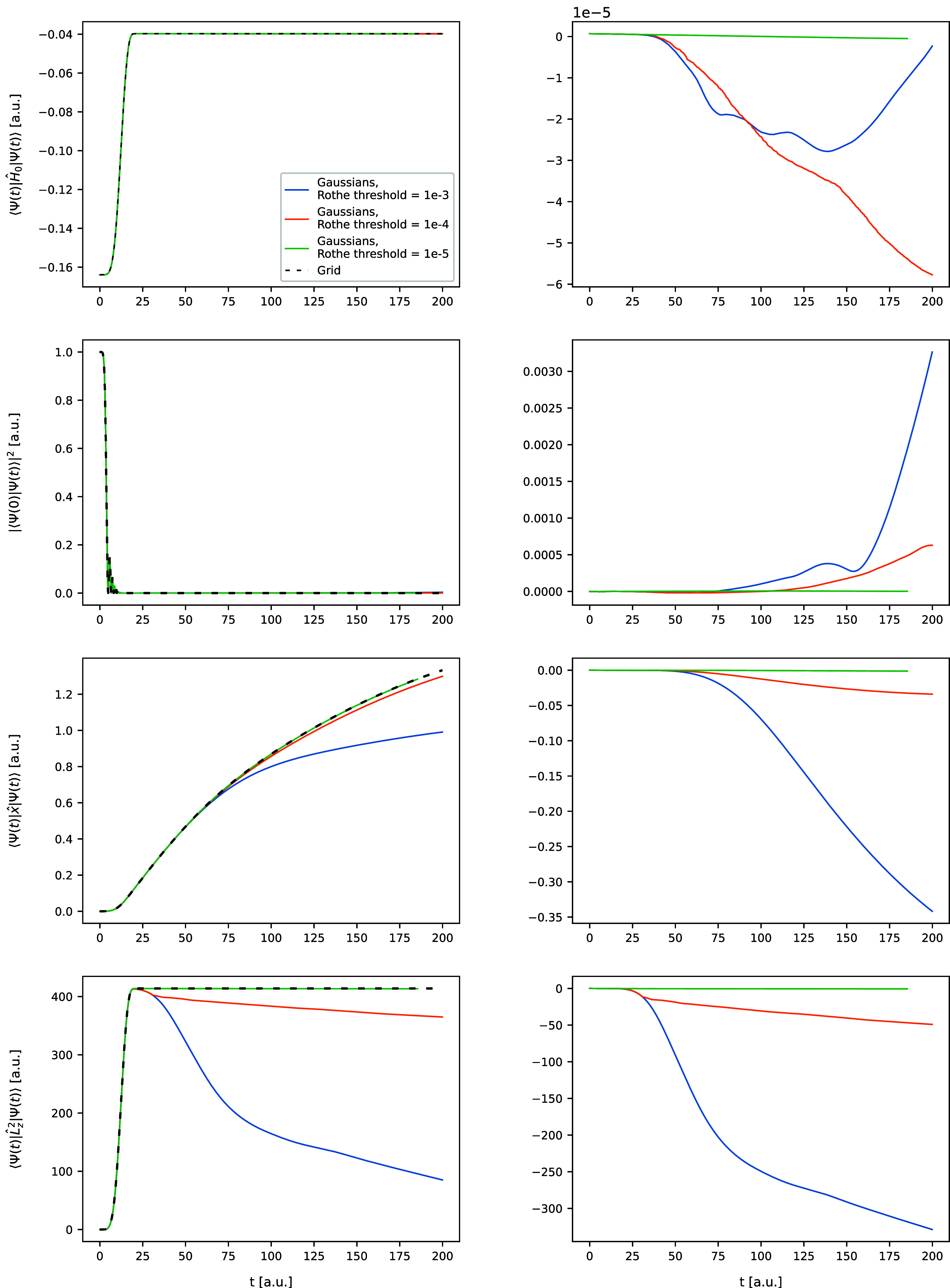
Same as Figure 1, but for the Morse model.

**Figure 3:**
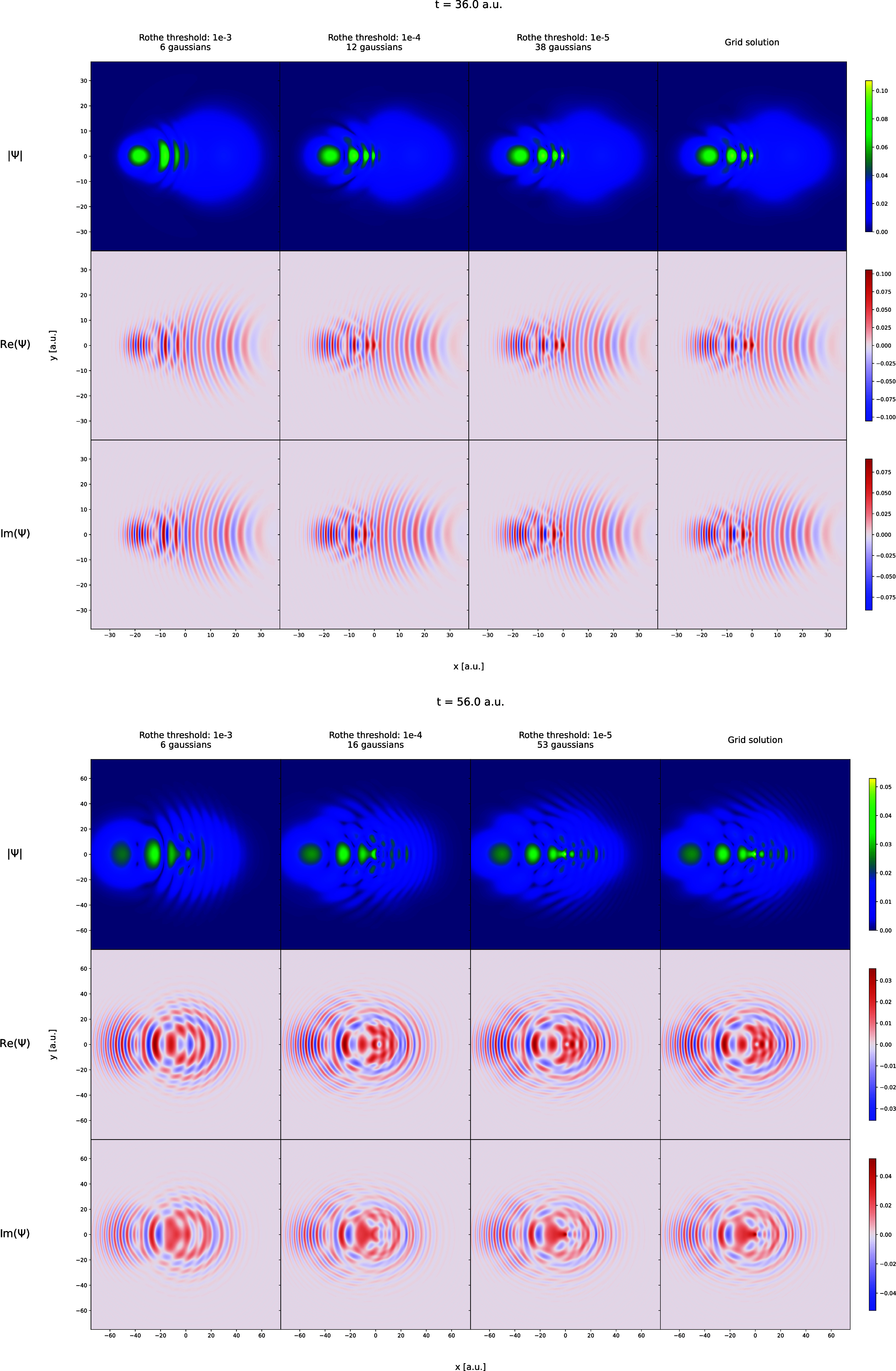
Wave packet, Ψ for the Coulomb model pictured for time equal to 36.0 and 56.0 au using plots of |Ψ|, Re­(Ψ), and Im­(Ψ). The wave packets shown in the first, second, and third columns of frames are obtained in the Rothe propagation using the accuracy threshold values of 10^–3^, 10^–4^, and 10^–5^, respectively. The last column of frames shows the wave packet obtained from the grid calculations.

**Figure 4:**
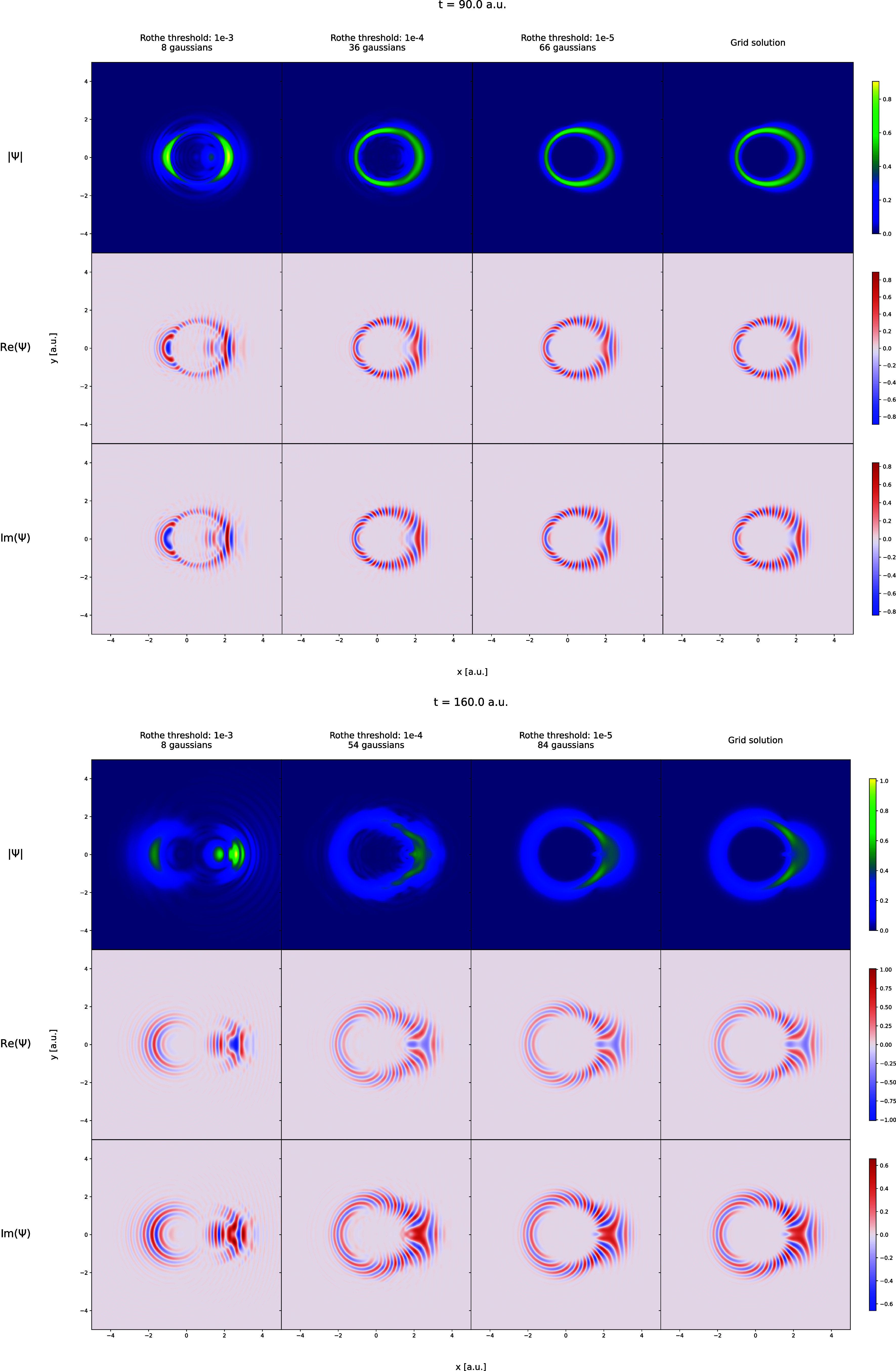
Wave packet, Ψ for the Morse model pictured for time equal to 90.0 and 160.0 au using plots of |Ψ|, Re­(Ψ), and Im­(Ψ). The wave packets shown in the first, second, and third columns of frames are obtained in the Rothe propagation using the accuracy threshold values of 10^–3^, 10^–4^, and 10^–5^, respectively. The last column of frames shows the wave packet obtained from the grid calculations.

**Figure 5:**
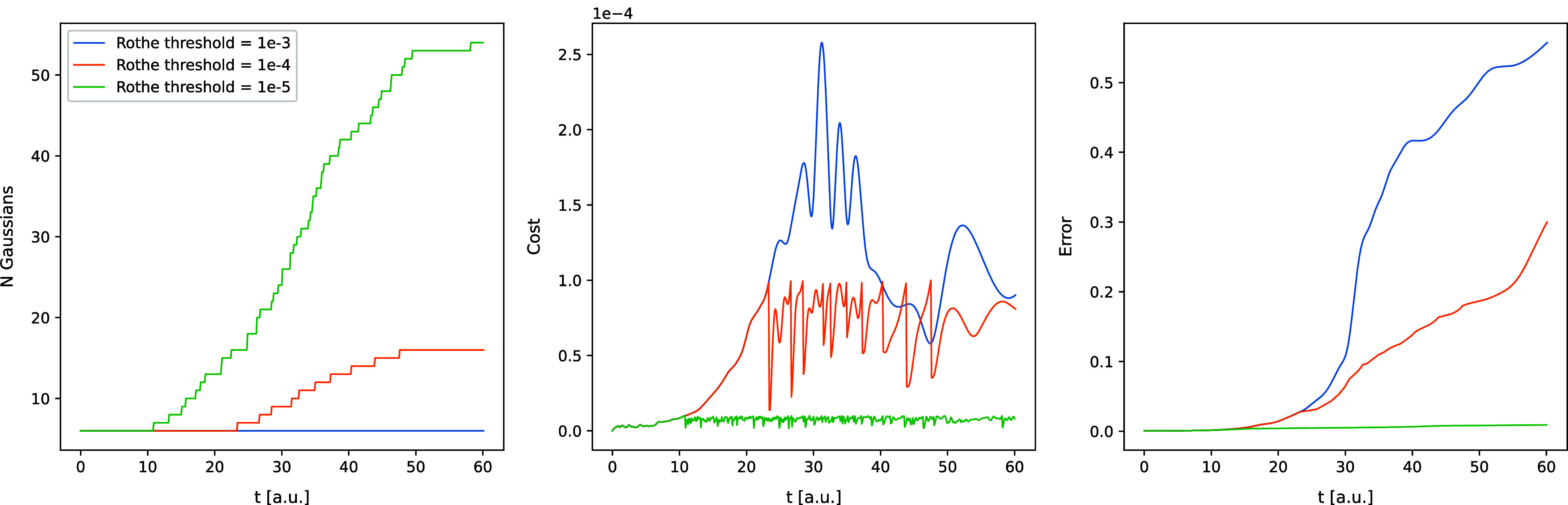
Results of the Rothe time propagation that employs FFECG basis functions for expending the wave packet for the Coulomb model. Time is on the horizontal axis in all plots. The leftmost plot shows the number of FFECGs in the basis set that is needed to achieve certain level of accuracy in the calculation as define by the value of the accuracy threshold, ϵ. The middle plot shows cost function variation with time for each of the three values of ϵ. The rightmost plot shows the error of the Rothe propagation with respect to the grid propagation.

**Figure 6:**
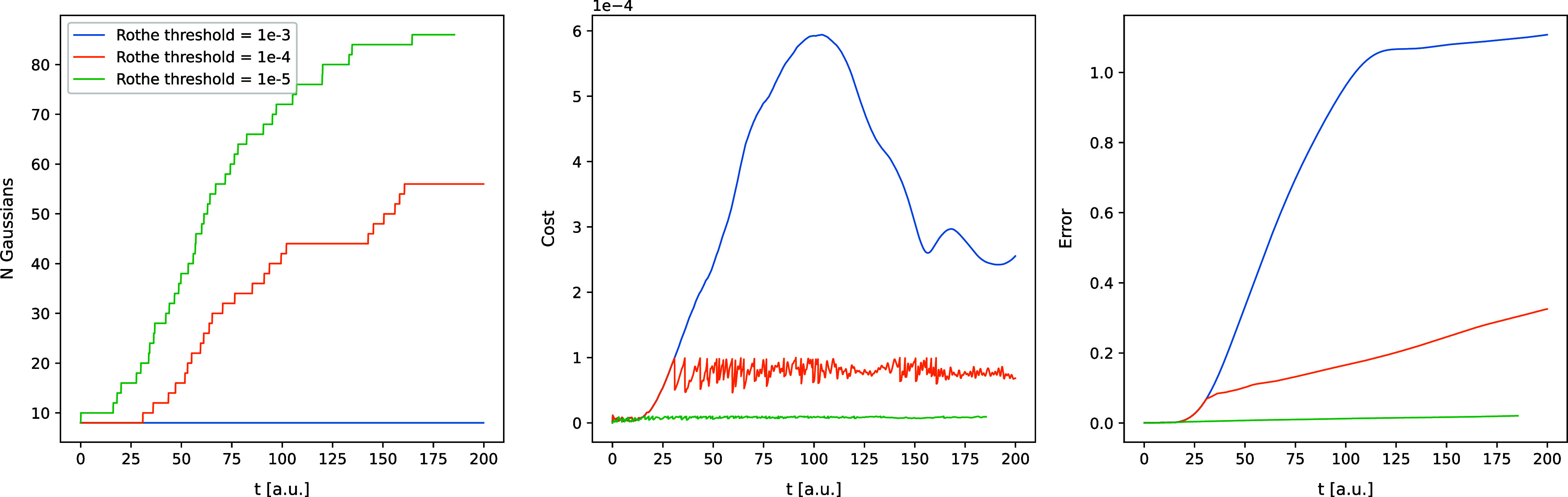
Same as Figure 5, but for the Morse model.

First and foremost, the simulations with the tightest adopted cost threshold ϵ for both models yield time-resolved observables that are virtually indistinguishable from those calculated using the grid-based approach. This is particularly evident in the plots of the observable errors (left columns of Figures 1 and 2), all of which consistently maintain a negligible level throughout the entire propagation periods. This consistency is further confirmed by comparing the wave function shapes in the two rightmost columns of Figures 3 and 4. This result is the main outcome of this work, as it directly confirms that the Rothe propagation algorithm – and, by extension, the reformulation of the TDSE as an optimization problem – is fundamentally equivalent to traditional time-discretization approaches which can all be seen as based on the Magnus formalism. Furthermore, it reinforces the conclusions of our previous work, that the FFECG basis set can perform on par with spatial grids. These results are even more striking when considering that the FFECG propagations and the grid-based propagations are performed completely independently, without any exchange of information about the wave function shape neither during the ground-state computation nor at any point during the propagation. However, as expected, this excellent agreement comes at a cost: a tighter cost threshold leads to a rapid increase of the basis set size. By the end of the simulations with threshold 10^–5^, the number of FFECGs exceeds 50 for the Coulomb model and 80 for the Morse model, impacting the CPU-time use per time step.

Loosening the threshold to 10^–4^ significantly reduces the final number of Gaussians needed – approximately 15 for the Coulomb model and 55 for the Morse model – while still producing results very close to the grid-based ones in terms of both the observables and the wave functions. However, some regions of lesser agreement with the grid results can be observed in Figure 3. For instance, in the snapshots from the Coulomb model trajectory, this simulation variant fails to reproduce the wave function maximum near the nucleus when the wave function becomes highly dissipated.

Finally, the results for the simulations with threshold 10^–3^ are best described as qualitatively or semiquantitatively correct rather than quantitatively correct. Noticeable deviations from the grid curves are apparent for the observables in Figures 1 and 2, and for the wave function shapes in Figures 3 and 4. The Rothe results only roughly approximate the grid results. Nevertheless, the overall shapes of the curves are preserved in most observables, with only two exceptions, the ⟨*x*⟩ and ⟨*L*_*z*_^2^⟩ trajectories for the Morse model in Figure 2. The discrepancy in ⟨*x*⟩ in the Morse model can be attributed to the nature of the dissociation. Due to the high nuclear masses, even when the molecule is excited to very high energies, as occurs in the simulation, the majority of the wave function remains well-localized, while only a small portion undergoes scattering. Describing both components simultaneously using a small number of Gaussians proves challenging, as seen in the leftmost column of Figure 4. This leads to the dipole moment stabilizing at a smaller value than in the reference simulation. The insufficient number of basis functions also accounts for the disagreement in the angular-momentum-projection expectation value. In the calculations, we use only *S*-type FFECGs (not multiplied by spherical harmonics), meaning that the angular component of the wave function arises solely from mixing between the plane-wave components of the Gaussians. If the basis set contains too few frequencies (*q*_*x*_ and *q*_*y*_ values), the resulting wave function may struggle to incorporate higher angular momenta. This is evident in Figure 2, where ⟨*L*_*z*_^2^⟩ starts to rapidly decrease immediately after the laser pulse ends. This suggests that the wave function undergoes a reflection from the basis set boundary, similar to effects observed in HHG calculations with Gaussian basis sets.^60–63^ On the other hand, the trajectories of ⟨*H*_0_⟩ and ⟨Ψ_0_ |Ψ­(*t*)⟩ in the Morse model, as well as the trajectories of all four observables in the Coulomb model, remain within a few percent error from the reference grid curves. Notably, for both models, the threshold of 10^–3^ did not necessitate any expansion of the basis set, i.e., the entire propagation is conducted using only the initial six Gaussians for the Coulomb model and eight Gaussians for the Morse model. Of course, in every set of the propagations, the nonlinear parameters of the Gaussians are reoptimized. As a result, the propagations took only a few hours to complete. Given such low computational complexity, the observed shortcomings may be considered acceptable trade-offs.

Overall, the basis expansion rate (the average number of the added functions per time step) is noticeably higher for the Morse model than for the Coulomb model. This indicates that the ro-vibrational dynamics of the Morse model is inherently more difficult to describe using Gaussians. This observation aligns with our previous findings when fitting the numerical time-dependent wave functions with FFECGs. This can be attributed to the aforementioned necessity of simultaneously describing both the well-localized, annular-shaped part of the wave function and the highly diffuse scattering part. Additionally, the maximum density of the second nucleus in the Morse model is shifted away from the reference nucleus by a certain distance, making it more challenging to represent using Gaussians. Another factor of significance is that, in the Morse simulation, two Gaussians are added at a time. However, when these results are translated to real time, the difference diminishes or even shifts in favor of the Morse model. For instance, at *t* = 45 au, with the threshold of 10^–4^ the wave functions consist of 15 and 14 FFECGs for the Coulomb and Morse models, respectively. For the same time, with the threshold of 10^–5^, the wave functions consist of 48 and 32 FFECGs for the Coulomb and Morse models, respectively. This suggests that the more complex nature of the nuclear dynamics is somewhat offset by the slower motion of the nuclei due to their higher masses. While this is only a rough estimation – given the significantly different shapes of the laser pulses applied to both models and the dynamics they induce – it may be seen as a promising indication for future simulations involving both the nuclear and electronic motions, as it implies that the basis of the electronic and nuclear wave functions should be augmented at a similar rate.

## Conclusions

The Rothe propagation method is used to obtain solution for the time-dependent Schrödinger equation for two 2D model systems that represent features which appear in non-Born–Oppenheimer quantum-dynamics time-propagation of the wave packet representing a diatomic neutral molecule interacting with a short intense laser pulse. In non-Born–Oppenheimer dynamics, all particles, i.e., the nuclei and electrons are treated on an equal footing. In the Rothe propagation, the wave packet representing the system is expanded in terms of the general fully flexible 2D explicitly correlated Gaussians (FFECGs) with complex exponential parameters. The Rothe time-propagation procedure, also known as the adaptive method of time layers,^64^ is based on a semidiscrete time-dependent Schrödinger equation using the implicit midpoint rule, and variationally minimizing its residual. The minimization is performed with respect to the linear expansion coefficients in the linear expansion of the wave packet in terms of FFECGs and with respect to the FFECG nonlinear parameter. The procedure involves growing the basis set of the Gaussians to provide a uniformly good representation of the time-evolving wave packet. The Rothe results are compared with the results obtained from the grid time propagation. Besides comparing the energy values and the wave functions, other properties are also calculated and used to assess the quality of the Rothe results. The comparison of the results show reliable performance of the Rothe method in solving the time-dependent Schrödinger equation for both model systems considered in this work.

Finally, this work represents a preliminary step in the application of FFECGs to describe the coupled electronic-nuclear dynamics in atomic and molecular systems. In the future work involving FFECGs and the non-BO nuclear-electronic quantum dynamics, the Rothe method will be employed to propagate the wave packet representing a real chemical system. The Rothe approach is a viable alternative to the standard real-time propagation techniques based on the McLachlan or time-dependent variational principles that involve numerically solving very stiff ordinary-differential equations. The FFECG optimization protocol developed in this work to minimize the Rothe functional with respect to the linear and nonlinear parameters of the FFECGs will be applied in our future work to time propagate FFECG wave packet representing real atomic and molecular systems. In the propagation, we will employ analytical gradients of the Rothe functional determined with respect to the FFECG nonlinear parameters, to aid the optimization. The approach involving the analytical energy gradient in variational ECG calculations of ground and excited stationary states has been successfully implemented in calculations for small atoms and molecules. The use of the gradient has significantly expedited the functional minimization and enabled to obtain non-BO energies and the corresponding wave functions whose accuracy by far exceeds the results obtained by other techniques. Implementation of the gradient in the Rothe method will require derivation of new algorithms to calculate gradient matrix elements. As in the case of the variational algorithm in the calculations of stationary states, this can be done using the matrix differential calculus. An effective implementation of the Rothe method to real chemical systems will also require highly efficient parallelization and vectorization, as well as implementation of GPU-enabled strategies.
